# Subclinical hearing loss in women with polycystic ovary syndrome: a case-control study using extended high-frequency audiometry

**DOI:** 10.1007/s00405-026-10304-x

**Published:** 2026-05-21

**Authors:** Emel Tahir, Asuman Küçüköner, Ulaş Çoban

**Affiliations:** 1 Ondokuz Mayıs University School of Medicine Department of Otolaryngology, Samsun, Turkey; 2 Ondokuz Mayıs University School of Medicine Department of Obstetrics and Gynecology, Samsun, Turkey

**Keywords:** Polycystic ovary syndrome, Sensorineural hearing loss, Extended high-frequency audiometry, Cochlea

## Abstract

**Objective:**

To determine whether women with polycystic ovary syndrome (PCOS) exhibit hearing loss and if so, to identify the specific frequencies involved using extended high-frequency audiometry.

**Methods:**

This single-centre, prospective, cross-sectional case-control study included 39 women with PCOS (diagnosed according to the Rotterdam criteria) and 39 age-matched controls. All participants underwent otoscopic examination, tympanometry, conventional pure-tone audiometry (250–8,000 Hz), and extended high-frequency audiometry (up to 12,500 Hz). Speech reception thresholds (SRT) and speech discrimination (SD) scores were also tested.

**Results:**

No statistically significant between-group differences were detected at 1,000–8,000 Hz. Women with PCOS demonstrated significantly elevated thresholds at 250 Hz and 500 Hz in both ears (*p* < 0.05), and at 11,200 Hz (right: *p* = 0.004; left: *p* < 0.001) and 12,500 Hz (left: *p* = 0.008). SRT was significantly higher in the PCOS group in the right and left ears. (*p* = 0.020 and *p* = 0.002 respectively). Speech discrimination scores were significantly lower in the PCOS group bilaterally (right: *p* = 0.036; left: *p* = 0.005).

**Conclusion:**

Women with PCOS exhibit detectable but subclinical alterations in extended high-frequency hearing and speech performance despite normal conventional audiometric thresholds. The frequency-specific pattern of threshold elevation is consistent with cochlear involvement potentially mediated by metabolic, inflammatory, and hyperandrogenic mechanisms. Extended high-frequency audiometry may serve as a sensitive tool for detecting early auditory dysfunction in women with PCOS.

## Introduction

Polycystic ovary syndrome (PCOS) affects approximately 5–10% of women of reproductive age, making it one of the most common endocrine disorders in this group [1, 2]. Beyond its defining reproductive features—oligo/anovulation, hyperandrogenism, and polycystic ovarian morphology—the 2023 International Evidence-Based Guideline recognizes substantial metabolic and cardiometabolic risk associated with the syndrome [2]. Insulin resistance, dyslipidemia, and chronic low-grade inflammation are well-documented accompaniments that drive systemic vascular changes and increase long-term health risks [2, 3]. Collectively, these metabolic features create a systemic milieu that may affect organs beyond the reproductive system—including the cochlea.

The cochlea is vulnerable to systemic metabolic and vascular insults. Epidemiological data link diabetes mellitus to a significantly elevated risk of sensorineural hearing loss [4, 5]. Korean population-based data further demonstrate that metabolic syndrome is independently associated with hearing impairment [6]. Cochlear vulnerability in these conditions is attributed to oxidative stress and endothelial dysfunction—mechanisms that are also cardinal features of PCOS pathophysiology [3]. The basal cochlear turn, which encodes high-frequency sounds, is especially susceptible to such insults due to its high metabolic demand and terminal microvascular supply. Early cochlear injury therefore tends to emerge first at higher frequencies, and extended high-frequency audiometry has been proposed as a sensitive tool for detecting these preclinical changes before they appear within the conventional audiometric range [7].

Several studies have specifically examined cochlear function in women with PCOS. Oghan and Coksuer [8] were among the first to document elevated high-frequency hearing thresholds in PCOS relative to healthy controls. Subsequent studies extended these findings, demonstrating threshold elevations within the 8000–14,000 Hz range [9, 10]. Notably, Turan et al. [10] also identified positive correlations between extended high-frequency thresholds and markers of hyperandrogenism, including the free testosterone index and hirsutism scores, suggesting a potential hormonal contribution to auditory alterations. More recently, Apeksha et al. (2022) observed both elevated high-frequency thresholds and reduced speech perception in noise performance in women with PCOS [11]. A 2025 systematic review and meta-analysis further supported these findings, demonstrating a significant association between PCOS and frequency-specific sensorineural hearing loss, with greater threshold elevations observed at higher frequencies [12].

The available evidence is nonetheless constrained by limited sample sizes and considerable methodological inconsistency, particularly in the definition of high-frequency ranges, calibration standards, and audiometric protocols. Furthermore, whether EHFA provides meaningful diagnostic value beyond conventional pure-tone testing specifically in women with PCOS has not been formally established.

This case–control study therefore aimed to assess pure-tone audiometric thresholds across a broad frequency range—including extended high frequencies—in women with PCOS relative to age-matched healthy controls, and to evaluate whether EHFA captures auditory alterations not detected by conventional testing. We hypothesized that PCOS would be associated with frequency-specific threshold elevations despite normal conventional audiograms.

## Materials and methods

### Patients

Ethical approval was obtained from the Institutional Ethics Committee (protocol no. 2022/560). The study was conducted in accordance with the Declaration of Helsinki. Prior to enrolment, all participants received a verbal and written explanation of the study objectives and the planned audiological procedures. Written informed consent was obtained from each individual before inclusion. This single-centre, prospective, cross-sectional study enrolled 78 participants: 39 women with PCOS and 39 age-matched healthy controls.Cases 3were women referred from the obstetrics and gynaecology outpatient clinic. Diagnosis was established by a gynaecologist using the Rotterdam criteria [13], in line with the 2023 International Evidence-based Guideline recommendations [2]. After excluding other causes of hyperandrogenism, PCOS was confirmed when at least two of the following three criteria were satisfied: (1) clinical and/or biochemical hyperandrogenism, (2) oligo/anovulation, and (3) polycystic ovarian morphology (PCOM) on ultrasound.

Controls were recruited on a voluntary basis from hospital staff and university students. Eligibility required the absence of any current or prior PCOS diagnosis and no known endocrine disorder.

Both groups were required to be aged 18–45 years, with no active ear pathology or previous otological surgery. Exclusion criteria for both groups included pre-existing hearing loss, chronic or active middle ear disease, ototoxic drug use, current pregnancy, concurrent endocrine conditions (including thyroid disease or diabetes mellitus), and any systemic or neurological disorder that could compromise audiometric test reliability.

## Audiological assessment

All subjects underwent comprehensive ear examinations to exclude abnormalities that might affect hearing, such as a perforated tympanic membrane or other middle-ear problems. An otolaryngologist performed an otoscopic examination, while an expert audiologist conducted audiometry and tympanometry examinations. All 78 subjects exhibited normal tympanic membranes as determined by otoscopic testing. All subjects exhibited normal middle ear function as identified by conventional immittance audiometry (GSI TympStar Pro, Midlefart, Denmark). Subsequently, tympanometry (Madsen Zodiac Diagnostic, model 1096), conventional pure-tone audiometry, and high-frequency audiometry (Interacoustics A/S, Denmark) were performed within a frequency range of 125 Hz to 16 kHz in a double-walled soundproof chamber. Conventional audiometry utilised Radioear B7 and Radioear 3045 headphones (RadioEar, Midlefart, Denmark), while high-frequency audiometry employed Radioear DD450 headphones. All audiometry testing equipment was calibrated in compliance with ISO 389-5 and EN ISO 8253-1:2010 regulations, as well as EN ISO 266:1997 regarding preferred frequencies.

Monosyllabic terms were employed to evaluate speech discrimination (SD). The speech reception threshold was tested using spondaic words, which are two-syllable words with equal stress on each syllable. The SRT was determined as the decibel hearing threshold below which the patient could not identify two-syllable words.

## Statistical method

Statistical analyses were carried out in R (version 4.4.1). Distribution normality was assessed using the Shapiro–Wilk test. Because audiometric threshold data were non-normally distributed, group comparisons were performed with the Mann–Whitney U test. Results are expressed as median (minimum–maximum) with mean rank values. Effect sizes are reported as Cohen’s d. Sample size was determined prior to data collection using G*Power (v3.1.7). An a priori power analysis at an effect size of d = 0.796 and α = 0.05 indicated that 39 participants per group would achieve 96% statistical power [8]. The significance level was set at *p* < 0.05.

## Results

When the groups were compared for demographic characteristics, no statistically significant difference was found between the control and patient groups in age (median age: 25 in the control group, 26 in the patient group; MR: 37.8 vs. 41.2; *p* = 0.513).

### Extended audiometry

#### Low frequencies (250–500 Hz)

When pure-tone thresholds at low frequencies were examined, the patient group showed higher thresholds than the control group.

At 250 Hz, the median value in the right ear was 10 dB (MR = 32.6) in the control group and 10 dB (MR = 46.4) in the patient group, with a statistically significant difference between the groups (*p* = 0.007). In the left ear, the median value was 5 dB (MR = 32.8) in the control group and 10 dB (MR = 46.2) in the patient group; the difference was statistically significant (*p* = 0.009).

At 500 Hz, the median value in the right ear was 10 dB (MR = 34.4) in the control group and 10 dB (MR = 44.6) in the patient group, with a significant difference between the groups (*p* = 0.048). In the left ear, the median was 5 dB (MR = 32.6) in the control group and 10 dB (MR = 46.4) in the patient group; the difference was statistically significant (*p* = 0.007).

### Mid and mid-high frequencies (1000–6000 Hz)

When evaluating the mid- and mid-high-frequency bands, no significant difference was found between the control and patient groups in either the right or left ear at 1000–6000 Hz. The median hearing thresholds for both groups at these frequencies were 10 dB, and statistical analyses showed no significant difference between groups (*p* > 0.05 for all comparisons).

## High frequencies (8000–12.500 Hz)

At 8000 Hz, the median hearing threshold in the right ear was 10 dB in both the control group (MR = 39.9) and the patient group (MR = 39.1), with no statistically significant difference between the groups (*p* = 0.885); similarly, in the left ear, median thresholds were comparable between the patient (MR = 39.6) and control (MR = 39.4) groups, and no significant difference was observed (*p* = 0.956). At 9000 Hz, the median right ear threshold was 15 dB in the control group (MR = 35.9) and 20 dB in the patient group (MR = 43.1), although this difference did not reach statistical significance (*p* = 0.159); in the left ear, both groups demonstrated a median threshold of 25 dB, with comparable mean ranks (patient: MR = 38.7; control: MR = 40.3) and no significant difference between the groups (*p* = 0.764). At 11,200 Hz, the median value in the control group was 15 dB (MR = 32.2) in the right ear. In contrast, in the patient group, it was 20 dB (MR = 46.8), revealing a statistically significant difference between the groups (*p* = 0.004). The median in the left ear was 10 dB (MR = 29.5) for the control group and 20 dB (MR = 49.5) for the PCOS group, with a statistically significant difference (*p* < 0.001).

At 12,500 Hz, the median values for the right ear were 15 dB in both the control and patient groups, with comparable MR values (39.6 vs. 38.5; *p* = 0.831). Conversely, in the left ear, the median value was 15 dB (MR = 32.7) in the control group and 25 dB (MR = 46.3) in the patient group, with a statistically significant difference between the groups (*p* = 0.008).

**Fig. 1:**
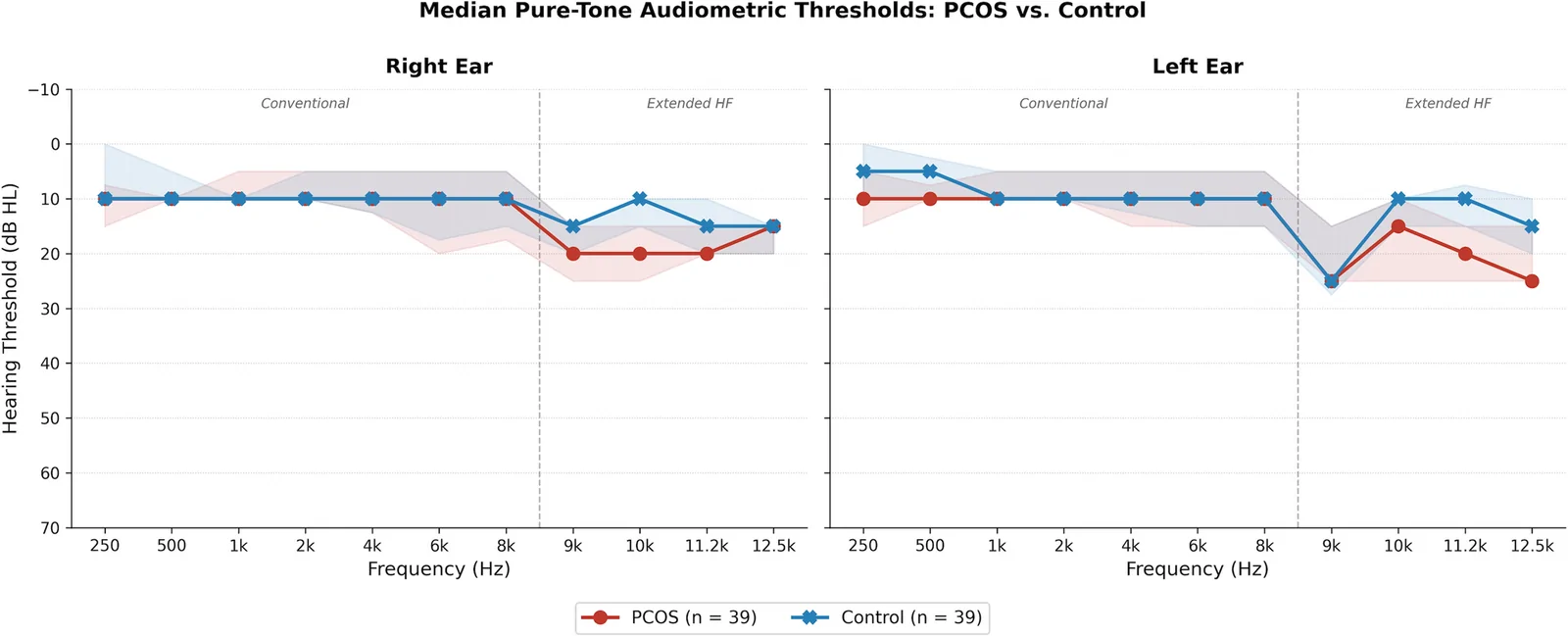
Figure 1. Median pure-tone audiometric thresholds across conventional (250–8,000 Hz) and extended high-frequency (9,000–12,500 Hz) ranges in women with polycystic ovary syndrome (PCOS) and healthy controls for the right ear (**A**) and left ear (**B**). Circles (●) represent the PCOS group (n = 39); crosses (✕) represent the control group (n = 39). Shaded areas indicate the interquartile range (IQR)

Hearing thresholds at conventional and high frequencies were compared between the patient and control groups; detailed median values, mean ranks, and statistical comparisons are summarised in Table 1. The frequency-specific pattern of threshold elevation is illustrated in Fig. 1.

**Table 1:** Comparison of audiometric thresholds and speech-related measures between patient and control groups

Frequency	Control (dB HL)	Patient (dB HL)	U	*p*	d*
R250	10 (0: 20) / 32.6	10 (0: 20) / 46.4	-2.708	**0.007** ^x^	0.644
L250	5 (0: 40) / 32.8	10 (0: 20) / 46.2	-2.598	**0.009** ^x^	0.616
R500	10 (0: 20) / 34.4	10 (5: 20) / 44.6	-1.979	**0.048** ^x^	0.460
L500	5 (0: 35) / 32.6	10 (0: 20) / 46.4	-2.678	**0.007** ^x^	0.637
L1000	10 (0: 15) / 39.1	10 (0: 20) / 39.9	-0.140	0.889^x^	0.032
R1000	10 (0: 20) / 40.4	10 (0: 20) / 38.6	-0.345	0.730^x^	0.078
R2000	10 (0: 20) / 39.5	10 (0: 20) / 39.5	0.000	1.000^x^	0.000
L2000	10 (0: 20) / 40.4	10 (0: 20) / 38.6	-0.360	0.719^x^	0.082
R4000	10 (0: 35) / 39.5	10 (0: 35) / 39.5	0.000	1.000^x^	0.000
L4000	10 (0: 25) / 38.9	10 (0: 25) / 40.1	-0.220	0.826^x^	0.050
L6000	10 (0: 30) / 39.4	10 (0: 35) / 39.6	-0.025	0.980^x^	0.006
R6000	10 (0: 40) / 39.1	10 (0: 515) / 39.9	-0.140	0.889^x^	0.032
R8000	10 (0: 45) / 39.9	10 (0: 85) / 39.1	-0.145	0.885^x^	0.033
L8000	10 (0: 25) / 39.4	10 (0: 40) / 39.6	-0.055	0.956^x^	0.012
R9000	15 (10: 80) / 35.9	20 (10: 80) / 43.1	-1.409	0.159^x^	0.323
L9000	25 (5: 55) / 40.3	25 (10: 80) / 38.7	-0.300	0.764^x^	0.068
R10000	10 (5: 75) / 27.7	20 (10: 80) / 51.3	-4.597	**< 0.001** ^x^	1.219
L10000	10 (5: 75) / 31.7	15 (5: 45) / 47.3	-3.053	**0.002** ^x^	0.737
R11200	15 (5: 70) / 32.2	20 (10: 70) / 46.8	-2.853	**0.004** ^x^	0.683
L11200	10 (5: 70) / 29.5	20 (10: 45) / 49.5	-3.907	**< 0.001** ^x^	0.987
R12500	15 (10: 70) / 39.6	15 (10: 70) / 38.5	-0.214	0.831^x^	0.049
L12500	15 (5: 65) / 32.7	25 (10: 50) / 46.3	-2.638	**0.008** ^x^	0.626
RSRT	10 (5: 20) / 33.5	15 (10: 20) / 45.5	-2.334	**0.020** ^x^	0.548
LSRT	10 (5: 20) / 47.6	10 (0: 20) / 31.4	-3.173	**0.002** ^x^	0.770
RSD%	96 (88: 100) / 44.9	96 (68: 100) / 34.1	-2.099	**0.036** ^x^	0.489
LSD%	100 (92: 100) / 46.6	96 (88: 100) / 32.4	-2.783	**0.005** ^x^	0.664

^x^Mann-Whitney U Test; U=test statistics This table uses the median (minimum: maximum) / rank averages display; d*= Effect size; Calculated as Cohen’s d (2 levels)

Values are presented as median and mean ranks (MR). Hearing thresholds are expressed in dB HL

*dB HL* decibel hearing level; *MR* mean rank; *SD* speech discrimination; *SRT* speech reception threshold; R, right; L, left; %, percentage

## Speech audiometry

**Fig. 2:**
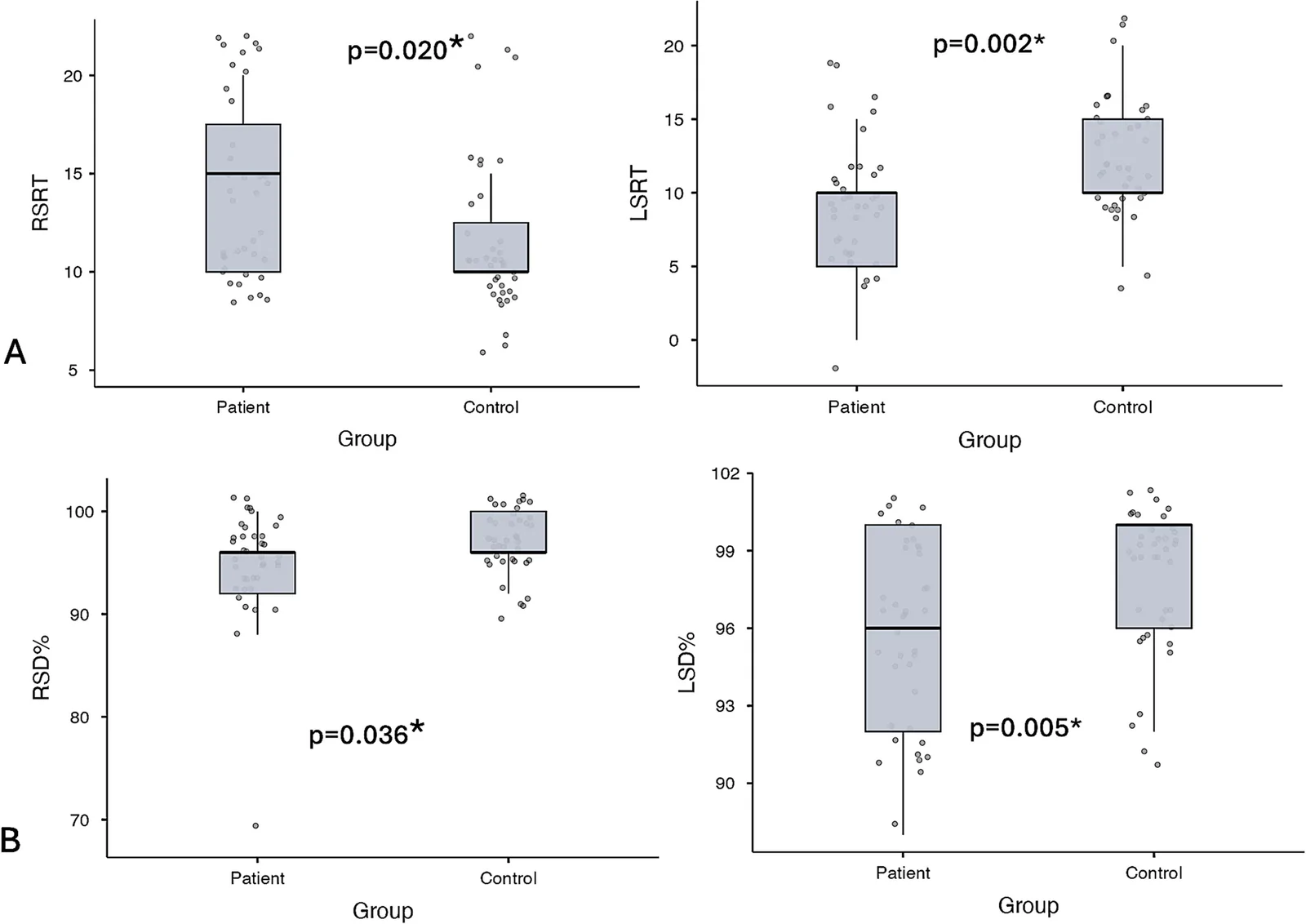
Boxplot comparison of speech reception thresholds (SRT/SD%) and speech discrimination scores (SD%) between the control and patient groups for the right and left ears. Boxes represent the interquartile range with the median indicated by the central line, while whiskers denote the minimum and maximum values. Between-group differences were assessed using the Mann–Whitney U test. Asterisks indicate statistically significant differences (p < 0.05). Higher values indicate poorer performance for SRT, whereas lower values indicate poorer performance for SD%.Abbreviations: SD, speech discrimination; SRT, speech reception threshold; R, right; L, left; %, percentage

The median speech reception threshold in the right ear was 10 dB (MR = 33.5) in the control group and 15 dB (MR = 45.5) in the patient group, with a statistically significant difference between the groups (*p* = 0.020). While the median values for the left ear were 10 dB in both the control and patient groups, the MR values were significantly higher in the control group (47.6 vs. 31.4), with a statistically significant difference (*p* = 0.002). (Fig. 2A).

At assessment of speech discrimination scores, the median score for the right ear was 96% (MR = 44.9) in the control group and 96% (MR = 34.1) in the patient group, revealing a statistically significant difference between the groups (*p* = 0.036). In the left ear, the median score was 100% (MR = 46.6) for the control group and 96% (MR = 32.4) for the patient group, with this difference also statistically significant (*p* = 0.005). (Fig. 2B).

## Discussion

This study documents measurable cochlear alterations in women with PCOS across a broad audiometric range. Significantly elevated thresholds were identified at 11,200 Hz and 12,500 Hz in the extended high-frequency domain, as well as at 250 and 500 Hz in the low-frequency range. Speech reception thresholds were raised and discrimination scores were reduced bilaterally. Crucially, these changes occurred in the context of entirely normal mid-frequency conventional audiograms, indicating that standard audiometry alone is insufficient to characterize the auditory profile of this population. Consistent with previous reports, we observed significantly elevated hearing thresholds in the extended high-frequency range, particularly at 11,200 Hz and 12,500 Hz. Oghan and Coksuer first described subclinical high-frequency sensorineural hearing loss (SNHL) in women with PCOS [8]. Subsequently, Kucur et al. demonstrated that extended high-frequency audiometry (EHFA) detects early cochlear impairment even when conventional frequencies remain within normal limits [9]. Similarly, Chen et al. reported that early auditory alterations in PCOS predominantly emerge in the 8–20 kHz range [14]. Our findings align with this pattern of preferential high-frequency vulnerability, reinforcing the hypothesis that the basal cochlear turn may be particularly sensitive to metabolic and vascular dysregulation associated with PCOS. The basal turn of the cochlea, which encodes high-frequency sounds, is particularly susceptible to vascular and metabolic disturbances due to its high metabolic demand and delicate microvascular supply [15]. EHFA exploits this anatomical vulnerability and provides a sensitive window into early cochlear injury before conventional threshold changes are apparent [16].

However, methodological heterogeneity across studies should be considered when interpreting cross-study comparisons. Differences in sample size, audiometric protocols, upper frequency limits, calibration procedures, and PCOS phenotype distribution may contribute to variability in reported threshold magnitudes. For example, Oghan and Coksuer [8] evaluated frequencies up to 8,000 Hz, whereas Kucur et al. [9] extended measurements to 20,000 Hz. Such methodological differences may partially explain discrepancies in frequency-specific findings.

Interestingly, our study also detected statistically significant differences at low frequencies (250–500 Hz). Given that prior studies emphasize predominant high-frequency sensitivity in PCOS [9, 12], systemic metabolic diseases have been associated with broader frequency effects. A systematic review and meta-analysis in type 2 diabetes mellitus demonstrated increased hearing thresholds across both low and high frequencies, with greater involvement at higher frequencies [4]. Similarly, population-based data from the Korea National Health and Nutrition Examination Survey identified a significant relationship between metabolic syndrome and hearing impairment [6]. These findings suggest that systemic metabolic dysregulation may affect cochlear physiology beyond strictly basal regions. Nevertheless, low-frequency thresholds are known to be more susceptible to environmental noise interference, subtle middle-ear pressure fluctuations, and test–retest variability. Therefore, although the observed differences were statistically significant, they should be interpreted cautiously and confirmed in future studies using repeated measurements and larger cohorts. Beyond pure-tone thresholds, our study extends the literature by incorporating speech discrimination (SD) performance. Although median SD scores remained within clinically acceptable limits, distributional analysis revealed a statistically significant shift toward lower performance in the PCOS group. Apeksha et al. [11] similarly reported reduced speech perception in noise among women with PCOS despite relatively preserved speech perception. These findings may reflect subtle cochlear or neural processing alterations that precede measurable threshold changes in conventional audiometry, a phenomenon previously described as hidden hearing loss or cochlear synaptopathy [17, 18]. The mechanisms linking PCOS to cochlear dysfunction are likely multifactorial. The stria vascularis—which maintains the electrochemical environment of the cochlear endolymph—depends on an intact microvasculature. Insulin resistance and endothelial dysfunction, both prevalent in PCOS [1, 2, 19], can impair cochlear microperfusion and compromise strial function. This impact falls disproportionately on the high-frequency basal region. Experimental data indicate that the cochlea is also a direct target of insulin signaling: insulin receptors and associated downstream proteins have been identified in auditory tissues, suggesting that insulin resistance may directly disrupt cochlear ion homeostasis [20]. Oxidative stress provides a complementary pathway. Cochlear outer hair cells and ribbon synapses are highly sensitive to reactive oxygen species, and oxidative injury to these structures underlies early synaptopathy—consistent with our speech performance findings [17, 18]. Chronic low-grade inflammation is another characteristic of PCOS [2, 3]. Elevated circulating inflammatory mediators [18], including C-reactive protein, have been documented in PCOS cohorts [9, 19], pointing to a systemic pro-inflammatory state that may amplify cochlear oxidative burden [3]. Androgen excess likely adds a further layer to cochlear vulnerability. Turan et al. reported positive correlations between EHFA thresholds and both the free androgen index and hirsutism scores [10], and Oghan and Coksuer also linked hyperandrogenism to hearing outcomes in PCOS [8]. The precise cochlear mechanisms of androgen action remain unclear, but vasoconstrictive and potentially direct ototoxic effects may act in concert with metabolic and inflammatory factors to accelerate basal cochlear deterioration.

These results do not support universal audiological screening in all women with PCOS. However, in those with pronounced metabolic features or significant hyperandrogenism, EHFA may uncover subclinical cochlear changes that conventional audiometry would miss. The inclusion of speech audiometry is also warranted, given the dissociation observed between conventional thresholds and speech reception and discrimination performance. Comprehensive audiological evaluation, including extended high-frequency testing and speech evaluation, may be informative in research settings or in patients reporting subtle auditory difficulties despite normal conventional audiograms.

Several limitations should be acknowledged. The cross-sectional design precludes causal inference. Objective auditory biomarkers such as otoacoustic emissions or electrophysiological measures were not included, limiting precise localization of dysfunction. In addition, PCOS phenotypes were not stratified, which may have masked differential vulnerability across subgroups.

Future longitudinal studies should evaluate auditory thresholds alongside metabolic and hormonal parameters, including insulin resistance indices, lipid profiles, inflammatory markers, and androgen levels. Phenotype-specific analyses may clarify whether particular metabolic or hyperandrogenic profiles confer greater auditory risk. Incorporation of speech-in-noise testing and patient-reported outcome measures may further elucidate the real-world functional impact of subtle auditory alterations in this population.

In conclusion, this study provides further evidence that women with PCOS exhibit measurable alterations in extended high-frequency hearing and subtle shifts in speech discrimination performance. Although these changes remain subclinical in magnitude, the observed frequency-specific pattern supports the hypothesis of early cochlear vulnerability potentially mediated by metabolic, inflammatory, and hormonal mechanisms. The auditory system may therefore represent a sensitive indicator of systemic dysregulation in PCOS and warrants further mechanistic and longitudinal investigation.

## Declarations

### Generative AI and AI-assisted technologies in the writing process

Grammarly (Grammarly Inc.) was utilized for grammar checking. After using these tools, the author(s) reviewed and edited the content as needed and take(s) full responsibility for the integrity and accuracy of the final manuscript.

### Data transparency

The data was submitted to the journal as supplementary material and share upon request. The data that support the findings of this study are openly available in Mendeley Data Storage as; Tahir, Emel (2026), “pcos hearing”, Mendeley Data, V1, doi: https://doi.org/10.17632/24vv4jmttn.1.
